# Mental Health and Wellbeing in Lithuanian Medical Students and Resident Doctors During COVID-19 Pandemic

**DOI:** 10.3389/fpsyt.2022.871137

**Published:** 2022-04-27

**Authors:** Agne Stanyte, Aurelija Podlipskyte, Egle Milasauskiene, Orsolya Király, Zsolt Demetrovics, Laurynas Ambrasas, Julius Burkauskas, Vesta Steibliene

**Affiliations:** ^1^Laboratory of Behavioral Medicine, Neuroscience Institute, Lithuanian University of Health Sciences, Palanga, Lithuania; ^2^Clinic of Psychiatry, Lithuanian University of Health Sciences, Kaunas, Lithuania; ^3^Institute of Psychology, ELTE Eötvös Loránd University, Budapest, Hungary; ^4^Centre of Excellence in Responsible Gaming, University of Gibraltar, Gibraltar, Gibraltar

**Keywords:** sleep, anxiety, depression, wellbeing, COVID-19, medical students, resident doctors

## Abstract

**Background:**

The coronavirus disease 2019 (COVID-19) pandemic has had a negative effect on mental health and subjective psychological wellbeing. One of the most affected population is medical students, reporting higher levels of depression, anxiety, sleep difficulties, and overall poorer wellbeing. However, the relationship between depression, anxiety, and sleep difficulties, and subjective psychological wellbeing has not been extensively researched in medical students in the context of COVID-19 pandemic. The aim of this study was to investigate the associations between depression, anxiety, and sleep quality, and subjective psychological wellbeing.

**Methods:**

In total, 524 medical students and resident doctors (78.6% female, mean age 24 ± 3 years old) participated in an online survey between December 2020 and February 2021. Participants completed the WHO—Five Wellbeing Index Questionnaire, the Pittsburgh Sleep Quality Index questionnaire, the Patient Health Questionnaire-9, and the Generalized Anxiety Disorder Assessment-7.

**Results:**

Multivariable logistic regression analysis showed that female participants’ worse subjective psychological wellbeing was associated with sleep difficulties [odds ratio (*OR*) = 2.39, 95% *CI* = 1.37–4.18, *p* = 0.002], higher depression (*OR* = 6.13, 95% *CI* = 3.46–10.88, *p* < 0.001), and anxiety symptoms (*OR* = 2.95, 95% *CI* = 1.66–5.22, *p* < 0.001). In male participants, analysis revealed an association between worse subjective psychological wellbeing and higher depression scores (*OR* = 9.94, 95% *CI* = 3.29–30.03, *p* < 0.001).

**Conclusion:**

Sex differences are an important factor to consider when evaluating subjective psychological wellbeing. Clinicians should be aware of significant contributors, such as sleep patterns anxiety, and depression, to subjective psychological wellbeing.

## Introduction

It is known that external life circumstances can have an impact on a persons’ subjective wellbeing (1, 2). The coronavirus disease 2019 [COVID-19; (3)] pandemic and restrictions, implemented to stop the spread of the virus, have caused a lot of disruption in today’s world. The COVID-19 pandemic can be described as a traumatic event, as one study in China found that the post-traumatic stress symptoms in the hardest-hit areas reached 7% (4). Individuals’ mental health and subjective psychological wellbeing may be jeopardized under such circumstances.

Early evidence suggests that the pandemic has had a negative and substantial effect on mental wellbeing. Research reports that subjective wellbeing is decreasing during the COVID-19 pandemic (5–9). Subjective psychological wellbeing is strongly linked to depression and anxiety symptoms (10) as well as sleep problems, such as insomnia (11), all of which have also been on the rise since the start of the COVID-19 pandemic (11–14).

When it comes to mental health and wellbeing, a significant amount of research has found differences between genders. In general, men are known to report higher levels of wellbeing compared with women (15). Across various different studies, women are more likely to have mental health problems compared with men (16–18), such as depressive symptoms, suicidal ideation, sleeping problems, and fatigue (18). Many risk factors for mental disorders, such as low educational level, low social support, inadequate income, and lack of physical, sexual, and psychological safety and security are more prominent in women compared with men (18, 19). Sex and gender differences can emanate from biomedical, psychosocial, or epidemiological reasons (19) and can impact the development of mental health disorders.

Differences in mental health and wellbeing have only escalated since the start of the pandemic. In a study conducted in the United Kingdom, women reported higher levels of depression, anxiety, insomnia, and had lower scores of wellbeing compared with men (20). Similar results were found in the United States (21), Poland (5), China (9), and Denmark (10). Overall, women have been found to be at a higher risk for psychological distress during the COVID-19 pandemic compared with men (22).

Nonetheless, men have also reported disturbances in their mental health, with depression symptoms being the most prevalent. A cross-sectional study of German students found major depressive syndromes were present for 36.8% of men, while anxiety syndromes were present for only 12% of men (23).

One of the most affected and vulnerable populations globally is medical students and resident doctors (24). Overall, medical students are known to have higher rates of depression, suicidal ideation, and are also less likely to seek help (25). The COVID-19 pandemic has only highlighted the problems in this population, with high levels of depression, anxiety (26–30), sleep problems, and decreased appetite (31). An Australian study found that the deterioration in mental wellbeing since the COVID-19 pandemic was reported by 68% of medical students (32). Another study reported that one in three students and medical residents showed symptoms of depression and anxiety, and almost 35% of students suffered from poor sleep quality (33). Such mental health problems can lead to burnout, which in the COVID-19 pandemic have become more prevalent in both medical students and resident doctors (34, 35). Most medical students perceive that the COVID-19 pandemic had a negative impact on their medical training, with pre-clinical medical students reporting the most difficulties in their academic and social life (36–39). The prevalence of stress, anxiety, and depression in medical students is significantly higher than in medical staff and community populations (40), making this population more vulnerable to the psychological effects of the COVID-19 pandemic to their psychological wellbeing.

However, the relationship between depression, anxiety, and sleep problems and subjective psychological wellbeing has not been researched in medical students and resident doctors in the context of the COVID-19 pandemic. In this study, we aimed to investigate the associations between depression, anxiety, and sleep quality and subjective psychological wellbeing. In the light of reviewed literature, we hypothesize that there will be significant sex differences in subjective wellbeing: anxiety and depression will be more important in the relationship among women, while the most important determinant for wellbeing in men will be depression.

## Materials and Methods

### Study Procedure

A cross-sectional study design was used to conduct the study within the Lithuanian University of Health Sciences (LUHS) during the COVID-19 pandemic period between December 2020 and February 2021. Participants—medical students and resident doctors—were invited to participate in the study *via* the official university mailing system and appropriate social media groups. They had to fill in an online survey available through Google Forms. To participate in the study, participants had to be older than or equal to 18 years old and provide online informed consent by ticking the appropriate answer “agree/disagree.” They received no incentives for participating in the study.

The study protocol was approved by the Bioethics committee of the LUHS (No. BEC-LSMU [R]-18) and the study was executed in accordance with the Declaration of Helsinki principles. More detailed description of study design and questionnaires used can be found in our initial study (41).

### Measures

The survey consisted of scales measuring general wellbeing (the WHO–Five Wellbeing Index (WHO-5) and Cronbach’s α was 0.88) (42, 43), sleeping patterns (Pittsburg sleep quality index (PSQI) and Cronbach’s α was 0.71) (44, 45), depression symptoms (Patient Health Questionnaire (PHQ-9) and Cronbach’s α was 0.84) (46), and anxiety symptoms (Generalized Anxiety Disorder Assessment (GAD-7) and Cronbach’s α was 0.91) (47, 48). Questions about socio-demographic characteristics, such as participants’ sex, age, living conditions, family situation, physical activity, academic achievements, and participation in academic classes were developed by the researchers.

### Statistical Analysis

Statistical analyses were performed with the Statistical Package for Science Software v.27 (SPSS, Chicago, IL). The comparisons between the socio-demographic characteristics and subjective psychological assessments between male and female study participants were completed using the two-tailed Student’s *t*-test and Fisher’s χ^2^-test. Multivariable logistic regression analyses were used to assess the relationship between wellbeing, sleeping patterns, depression, and anxiety symptoms, and the outcome variable was wellbeing. Two different models were created for male and female participants, adjusted for age, types of studies, and marital state. Regarding the sample size, a 10:1 ratio of cases to variables was used (49). The level of significance was set at *p* < 0.05.

## Results

Overall, 524 individuals participated in the study, of whom 65.6% were medical students and 34.4% were resident doctors. The mean age of participants was 23.7 ± 3.1 years, and the majority of the participants were women (*n* = 412). A detailed description of sample characteristics is presented in Table 1.

**TABLE 1:** Socio-demographic characteristics and subjective psychological assessments in study participants.

Characteristics	All, *n* = 524	Female, *n* = 412	Male, *n* = 112	t/χ^2^	Cohen’s *d*/Cramer’s *V*	*p*
Age, years; mean (*SD*)	23.7 (3.1)	23.4 (3.0)	24.5 (3.5)	−3.34	0.337	<0.001
**Types of studies, *n* (%)**				12.68	0.156	0.002
Pre-clinical medical student	206 (39.3)	174 (42.2)	32 (28.6)			
Clinical medical student	138 (26.3)	112 (27.2)	26 (23.2)			
Doctor resident	180 (34.4)	126 (30.6)	54 (48.2)			
**Living condition, *n* (%)**				0.40	0.028	0.525
Alone	174 (33.2)	134 (32.5)	40 (35.7)			
With partner/family members	350 (66.8)	278 (67.5)	72 (64.3)			
**Marital state, *n* (%)**				3.50	0.082	0.061
Single	266 (43.1)	169 (41.0)	57 (50.9)			
Married/Partnership	298 (56.9)	243 (59.0)	55 (49.1)			
**Smoking, *n* (%)**				3.37	0.080	0.066
Yes	112 (21.4)	81 (19.7)	31 (27.7)			
No	412 (78.6)	331 (80.3)	81 (72.3)			
Five wellbeing index WHO5, total score; mean (*SD*)	51.9 (18.9)	51.1 (18.6)	54.6 (19.7)	−1.76	0.183	0.078
WHO5 ≤ 50, *n* (%)	237 (45.2)	195 (47.3)	42 (37.5)	3.44	0.081	0.064
Global PSQI index, mean (SD)	6.6 (3.0)	6.1 (3.0)	6.0 (3.1)	0.36	0.033	0.723
PSQI ≥ 5, *n* (%)	339 (64.7)	271 (65.8)	68 (60.7)	0.99	0.043	0.320
PHQ-9, total score; mean (*SD*)	9.1 (5.7)	9.3 (5.8)	8.5 (5.6)	1.31	0.140	0.190
PHQ-9 ≥ 10, *n* (%)	218 (41.6)	174 (42.2)	44 (39.3)	0.32	0.025	0.575
GAD-7, total score; mean (*SD*)	7.4 (5.1)	7.7 (5.2)	6.4 (5.0)	2.33	0.255	0.020
GAD-7 ≥ 9, *n* (%)	179 (34.2)	149 (36.2)	30 (26.8)	3.44	0.081	0.063

*SD, standard deviation; WHO5, World health Organization—five wellbeing index; PSQI, Pittsburgh sleep quality index; PHQ-9, Patient health questionnaire; GAD-7, Generalized anxiety disorder assessment.*

In general, 45.2% of the participants reported a WHO-5 wellbeing score of less than 50, which indicates serious impairment of subjective psychological wellbeing (42). Sleep problems were reported by 64.7% of the participants. Higher depression symptoms were prevalent in 41.6% of the participants and anxiety symptoms in 34.2% of the participants.

Female participants were significantly younger (*t* = −3.34, 23.4 vs. 24.5, *p* < 0.001, medium effect *d* = 0.337), more of them were pre-clinical medical students (χ^2^ = 12.68, 42.2 vs. 28.6%, *p* < 0.002, small effect *V* = 0.156) and had a higher anxiety score (*t* = 2.33, 7.7 vs. 6.4, *p* = 0.020, small effect *d* = 0.255) than men (Table 1). In addition, we noticed a tendency that women were more likely to be married or in a partnership (χ^2^ = 3.50, 59.0 vs. 49.1%, *p* = 0.061), they were less likely to smoke (χ^2^ = 3.37, 19.7 vs. 27.7% *p* = 0.066) and had lower wellbeing (*t* = -1.76, 51.1 vs. 54.6, *p* = 0.078) compared with men. Due to significant differences between the sexes, further analysis was performed separately for men and women. Inter-correlation between study variables is presented in Supplementary Tables 1, 2.

Table 2 shows multivariable logistic regression analyses for women and men separately. Both models were adjusted for age, types of studies, and marital state. Multivariable logistic regression analysis for women showed that worse subjective psychological wellbeing is significantly associated with sleep difficulties [odds ratio (*OR*) = 2.39, 95% *CI* = 1.37–4.18, *p* = 0.002], higher depression (*OR* = 6.13, 95% *CI* = 3.46–10.88, *p* < 0.001), and anxiety symptoms (*OR* = 2.95, 95% *CI* = 1.66–5.22, *p* < 0.001). Multivariable logistic regression analysis for men revealed that worse subjective psychological wellbeing is significantly associated with higher depression scores (*OR* = 9.94, 95% *CI* = 3.29–30.03, *p* < 0.001).

**TABLE 2:** Multivariable logistic regression analyses for women and men.

		Outcome	
		Women	Men
	
		WHO 5 ≤ 50	WHO 5 ≤ 50
Predictors	*R*^2^(p)	0.333 (<0.001)	0.241 (<0.001)
PSQI ≥ 5 (1)	OR (95%CI) (p)	2.39 (1.37–4.18) (0.002)	0.94 (0.32–2.75) (0.910)
PHQ-9 ≥ 10 (1)	OR (95%CI) (p)	6.13 (3.46–10.88) (< 0.001)	9.94 (3.29–30.03) (<0.001)
GAD-7 ≥ 9 (1)	OR (95%CI) (p)	2.95 (1.66–5.22) (< 0.001)	1.27 (0.37–4.30) (0.609)
	Cohen’*f*^2^	0.499	0.331

*Multivariable: adjusted for age, types of studies, and marital state. WHO5, World Health Organization—five well-being index; PSQI, Pittsburgh sleep quality index; PHQ-9, Patient Health Questionnaire for depression symptom severity; and GAD-7, Generalized anxiety disorder assessment for anxiety symptom severity.*

## Discussion

Our study aimed to investigate the associations between depression, and anxiety, sleep quality and subjective psychological wellbeing. Our study revealed different associations of mental distress, such as anxiety and depression symptoms and subjective psychological wellbeing among men and women. In women, wellbeing was associated with sleep difficulties, depression, and anxiety symptoms. In comparison, in men, we found that wellbeing was associated only with depression. These results are in line with our hypotheses.

Comparing our results with other research, we can see some similarities between other countries. In a study conducted in Germany about students’ wellbeing, the researchers found that 72.2% of the participants had a WHO-5 wellbeing score of less than 50. Of the responders, 41.6% had indications of the major depressive syndromes (measured by PHQ-9), 20%—for generalized anxiety or panic syndromes (measured by GAD-7) (23). However, the researchers reported that students of medicine were less affected compared with students from other disciplines (23). Our study sample consisted only of medical students and residents, which could help explain the differences between samples in subjective psychological wellbeing scores. It is believed that medical students and resident doctors had more opportunities for social exchange during the COVID-19 pandemic due to the nature of work in healthcare and the fact that they could not work remotely, providing more opportunities for everyday social interactions and activities.

Nevertheless, mental health is in decline during the COVID-19 pandemic. A study from Turkey before the COVID-19 pandemic investigated depression and anxiety among medical students and found that 30.6% of them reported feeling depressive symptoms (measured by Beck depression inventory II) and 20.7% of students reported feeling anxiety (measured by Beck anxiety inventory) (50). Other studies before the COVID-19 pandemic have found similar results (51). Some differences between studies could arise from different forms of screening of depression and anxiety as well as from different cultural backgrounds. However, the trend of students reporting more depression than anxiety is still prevalent in both cases as well as in the current study.

Our study showed that subjective psychological wellbeing in women was associated with sleep difficulties, depression and anxiety. It is known that women consistently report poorer wellbeing compared with men (52) and such a divide is being made more apparent in the midst of the COVID-19 pandemic. Other sex-related factors, such as the unemployment rate (53) during COVID-19 pandemic might have contributed to this burden. Furthermore, the newest data suggest that the loss of non-parental child care and involvement in homeschooling were associated with more negative employment outcomes for mothers but not for fathers (54).

In our study, women consistently reported higher levels of depression, anxiety, and sleep difficulties, all of which were important to women’s overall wellbeing. This could be explained by the gender differences in mental disorders, where women are known to have a higher lifetime prevalence of mood and anxiety disorders (55). The differences can be partly explained by the potential role of the sex hormones acting as risk factors for depression (56), anxiety, stress–related, and trauma-related disorders (57).

Furthermore, we found that in men, the subjective psychological wellbeing was associated only with depression. No other research linking men’s depression to their subjective psychological wellbeing could be found for comparison. Gender roles may have implications for coping styles used in stressful situations. A cross-sectional study investigating the risk factors of psychological distress during the COVID-19 pandemic found that men were more likely to experience higher psychological distress compared with women, and were more likely to use negative coping styles, for example, emotion suppression (58). Psychological distress and negative coping styles can lead to deteriorated mental health, as it has been discovered that stressful life events have a predictive role for major depressive episodes in men (59). During the COVID-19 pandemic, men feel more stressed than before the pandemic. They employ unhealthy coping mechanisms to deal with their emotions, which in turn can lead to feelings of depression. Therefore, depression can have a bigger impact on men’s subjective psychological wellbeing compared with anxiety and sleep difficulties.

The study should be interpreted in the context of its design and limitations. First, the results depended on self-assessment data and cannot be generalized to a wider population, as only a small sample of medical students and resident doctors from Lithuania participated in the study. Second, the self-selected cross-sectional design prevents us from making implications with regards to causal effects between study variables. Third, the data for the study were collected during the COVID-19 pandemic period, therefore, we are unable to draw inferences on how the associations could possibly change after the pandemic is over. Last, we did not collect the COVID-19 related medical status of participants, isolation, or other variables related to the pandemic that could have had an impact on mental health and subjective psychological wellbeing.

It is known that women consistently report poorer wellbeing compared with men (52), however, not a lot of research has been dedicated to learn about sex differences. Understanding the differences and their causes could give us a more comprehensive understanding of psychological wellbeing as well as provide help in creating policies and programs designed to strengthen the wellbeing and mental health of men and women. This study highlighted the differences between associations of wellbeing, depression, anxiety, and sleep difficulties in men and women, showing that there are significant differences between the sexes and how they express subjective psychological wellbeing. Future research should concentrate on replicating these results in a wider population sample.

In conclusion, we found that in women, subjective psychological wellbeing is associated with sleep problems, depression, and anxiety. In men, subjective psychological wellbeing is associated only with depression.

## Data Availability Statement

The data analyzed in this study is subject to the following licenses/restrictions: Institutional agreement. Requests to access these datasets should be directed to VS (vesta.steibliene@lsmuni.lt).

## Ethics Statement

The studies involving human participants were reviewed and approved by the Bioethics committee of the LUHS (No. BEC-LSMU [R]-18). The patients/participants provided their written informed consent to participate in this study.

## Author Contributions

JB and VS designed the study. EM and LA collected and analyzed the data. AP performed statistical analyses. AS drafted the first manuscript. EM, JB, LA, AP, ZD, OK, and VS edited the manuscript. All authors contributed to the manuscript and approved the final version.

## Conflict of Interest

In the past several years JB has been serving as a consultant to Cogstate, Ltd. VS reported being a consultant to SignantHealth; received personal fees from Lundbeck, Sanofi, Servier, Johnson and Johnson, KRKA, grants from the Research Council of Lithuania. The remaining authors declare that the research was conducted in the absence of any commercial or financial relationships that could be construed as a potential conflict of interest.

## Publisher’s Note

All claims expressed in this article are solely those of the authors and do not necessarily represent those of their affiliated organizations, or those of the publisher, the editors and the reviewers. Any product that may be evaluated in this article, or claim that may be made by its manufacturer, is not guaranteed or endorsed by the publisher.
