# Supplementary material for: Mental Health and Wellbeing in Lithuanian Medical Students and Resident Doctors During COVID-19 Pandemic
Source: Front Psychiatry. 2022 Apr 27;13:871137. doi: 10.3389/fpsyt.2022.871137 (PMC9092277; doi:10.3389/fpsyt.2022.871137)
Supplement: Supplementary file 1 [file Data_Sheet_1.docx]

**Table 1S.** Pearson correlations for women

|  | WHO5 | PSQI | PHQ9 | GAD7 |
| --- | --- | --- | --- | --- |
| WHO5 | 1 |  |  |  |
| PSQI | –0.490  p<0.001 | 1 |  |  |
| PHQ9 | –0.667  p<0.001 | 0.636  p<0.001 | 1 |  |
| GAD7 | –0.559  p<0.001 | 0.463  p<0.001 | 0.754  p<0.001 | 1 |

*WHO5, World Health Organization – five well-being index; PSQI, Pittsburgh sleep quality index; PHQ-9, Patient Health Questionnaire; GAD-7, Generalized anxiety disorder assessment.*

**Table 2S.** Pearson correlations for men

|  | WHO5 | PSQI | PHQ9 | GAD7 |
| --- | --- | --- | --- | --- |
| WHO5 | 1 |  |  |  |
| PSQI | –0.295  p=0.002 | 1 |  |  |
| PHQ9 | –0.718  p<0.001 | 0.584  p<0.001 | 1 |  |
| GAD7 | –0.544  p<0.001 | 0.576  p<0.001 | 0.732  p<0.001 | 1 |

*WHO5, World Health Organization – five well-being index; PSQI, Pittsburgh sleep quality index; PHQ-9, Patient Health Questionnaire; GAD-7, Generalized anxiety disorder assessment.*
